# Wear Behavior of Conventionally and Directly Aged Maraging 18Ni-300 Steel Produced by Laser Powder Bed Fusion

**DOI:** 10.3390/ma14102588

**Published:** 2021-05-16

**Authors:** Kichang Bae, Dohyung Kim, Wookjin Lee, Yongho Park

**Affiliations:** 1 Dongam Division, Korea Institute of Industrial Technology, Yangsan 50623, Korea; kichbae@kitech.re.kr (K.B.); dhyungkim@kitech.re.kr (D.K.); 2 Department of Materials Science and Engineering, Pusan National University, Busan 46241, Korea

**Keywords:** maraging steel, additive manufacturing, laser powder bed fusion, selective laser melting, dry sliding wear

## Abstract

This study aims to explore the wear performance of maraging 18Ni-300 steel, fabricated via laser powder bed fusion (LPBF). The building direction dependence of wear resistance was investigated with various wear loads and in terms of ball-on-disk wear tests. The effect of direct aging heat treatment, i.e., aging without solution heat treatment, on the wear performance was investigated by comparing the wear rates of directly aged samples, followed by solution heat treatment. The effect of counterpart material on the wear performance of the maraging steel was studied using two counterpart materials of bearing steel and ZrO_2_ balls. When the bearing steel ball was used as the counterpart material, both the as-built and heat-treated maraging steel produced by the LPBF showed pronounced building direction dependence on their wear performance when the applied wear load was sufficiently high. However, when the ZrO_2_ ball was used as the counterpart material, isotropic wear resistance was reported. The maraging steel produced by the LPBF demonstrated excellent wear resistance, particularly when it was aging heat-treated and the counterpart material was ZrO_2_. The directly aged sample showed wear performance almost the same as the sample solution heat-treated and then aged, indicating that direct aging can be used as an alternative post heat treatment for tribological applications of the maraging steels produced by LPBF.

## 1. Introduction

Additive manufacturing (AM), a new manufacturing technology in which components are produced in a layer-by-layer manner, has been in the spotlight recently as an alternative to the traditional manufacturing methods for multiple industrial applications. AM enables direct manufacturing of 3D components from computer-aided design data. Among various AM techniques, the laser powder bed fusion (LPBF) process is one of the most commonly used techniques to produce metallic components with complex 3D shapes [1,2]. In this technique, the metallic powder feedstock is delivered to the workpiece by spreading flat powder layers in the powder bed during the process. A layer is consolidated by fusing a selective area of each powder layer by laser scanning. This process is repeated to produce 3D components.

Among various LPBF techniques, selective laser melting (SLM) is one of the most widely used methods to fabricate fully dense metallic components. Materials manufactured by the SLM process that have similar or better mechanical properties than the same materials manufactured by the traditional manufacturing method are reported [1,3]. Furthermore, wear-impact resistances of complex shapes produced by the SLM and by other manufacturing processes such as spark plasma sintering together are reported [4,5]. These studies highlight the advantages of using the SLM process for fabricating complex three dimensional shapes.

Maraging steels are a special class of low-carbon high-alloyed steels that offer hardenability through aging heat treatments via martensitic phase transformations. The maraging steels were applied to various mechanical components such as hot-work dies, bearing gear parts, pressure vessels, aircraft components, and rocket motor cases [6,7,8,9]. One advantage of using the maraging steel is its good hardenability without high contents of carbon. Because the maraging steel does not necessarily contain carbon for obtaining high strength or hardness, maraging steels can exhibit good mechanical properties with low-carbon or carbon-free states. The low-carbon maraging steel is easily weldable because no special care is required to avoid carbide or carbon segregation-related problems. This advantage facilitates its application in the LPBF process. Therefore, many studies have been conducted into the manufacture of maraging steel alloys through the LPBF processes, e.g., Kempen et al. [10] examined the effect of LPBF parameters and post-heat treatment on the mechanical properties of maraging steels. They demonstrated that the maraging steel produced by the LPBF can be hardened by performing aging heat treatment without solution heat treatment. Simson et al. [11] investigated the mechanical properties of maraging 18Ni-300 steel produced by the LPBF process. They reported that the hardness, tensile strength, and ductility of heat-treated maraging steel produced by the LPBF can be the same or higher than the alloy produced by the conventional method. Tan et al. [12] investigated the microstructural evolution, precipitation behaviors, and strengthening mechanism of the maraging steel fabricated via the LPBF process. They reported that the maraging steel produced by the LPBF has a fine microstructure with nano precipitates and amorphous phases in the as-built state because of extremely fast solidification during the LPBF process, which triggers the martensitic transformation and hardening of the alloy during the heat treatment.

When the maraging steels produced by the LPBF are used for tribological components such as tools, bearings, or dies, the wear resistance of materials is an important factor in determining the service life [13,14,15,16]. Therefore, the study of wear behavior under various conditions is required; however, only a few studies have been reported in the literature. For example, Yin et al. [17] investigated the effect of aging heat treatment conditions on the microstructure, mechanical properties, and tribological properties of the maraging steel fabricated by the LPBF. Tan et al. [18] show that aging heat treatment can remarkably improve the tribological performance of the maraging steel produced by the LPBF. However, there has been no study to characterize the building direction dependence of wear behavior for the maraging steel produced by the LPBF. Moreover, studies in the literature about the effects of various wear loads, heat treatment conditions, and counterpart material on the wear performance of maraging steel manufactured by the LPBF process are still insufficient.

This study explores the effects of several important parameters on the wear performance of the maraging steel produced by the LPBF for bearing gear housings where friction with metal materials occurs repeatedly. Low carbon AISI 18Ni-300 maraging steel was used for the study [19,20]. The effects of the wear loading direction with the LPBF building direction, heat treatment conditions, and counterpart materials were experimentally studied using various wear loads via ball-on-disk type wear tests. The wear during the operation of the bearing housings is due mainly to high hardness metal material that is in direct contact, and by small ceramic particles such as dusts or impurities. Therefore, two types of counterpart materials were selected: AISI 52100 steel and ZrO_2_.

## 2. Materials and Methods

In this study, the maraging 18Ni-300 steel samples examined were fabricated using an LPBF-type metal 3D printer (Sodick Co., Ltd., OPM 250L, Kyoto, Japan), equipped with a ytterbium fiber laser with a wavelength of 1070 nm and maximum laser output of 500 W (YLR-500-WC, IPG, Laser GmbH, Burbach, Germany) with a Gaussian beam distribution and a spot diameter of 200 µm. Gas-atomized maraging 18Ni-300 steel powder with an average particle diameter of ~40 μm (OPM Maraging, OPM Laboratory Co., Ltd., Kyoto, Japan) was used to fabricate the samples. The chemical composition of maraging 18Ni-300 steel powder used for the LPBF is given in Table 1. Table 2 lists the LPBF fabrication parameters that are used. The fabrication parameters were selected as conditions for the SLM process so that near fully dense specimens could be obtained. The LPBF was performed in a nitrogen environment with an oxygen content of <1% to prevent oxidation. A 90° rotation scanning strategy was used, i.e., the laser scanning lines were tilted by 90° between each layer. Certain samples underwent solution treatment for 2 h at 850 °C and then aged at 500 °C for 6 h (designated as HT850) to fully harden them to a martensitic structure [12,21]. Certain other specimens were directly aged without solution treatment at 450 °C for 6 h (designated as HT450). The heat treatments of the specimens were performed in a box-type laboratory furnace under atmospheric conditions. The specimens were wrapped in a protective heat-treatment foil to prevent oxidation of the sample surface. Note that additional details of the material, including the heat-treatment effect on the microstructure and tensile behavior, were reported in the previous study [22].

**Figure 1:**
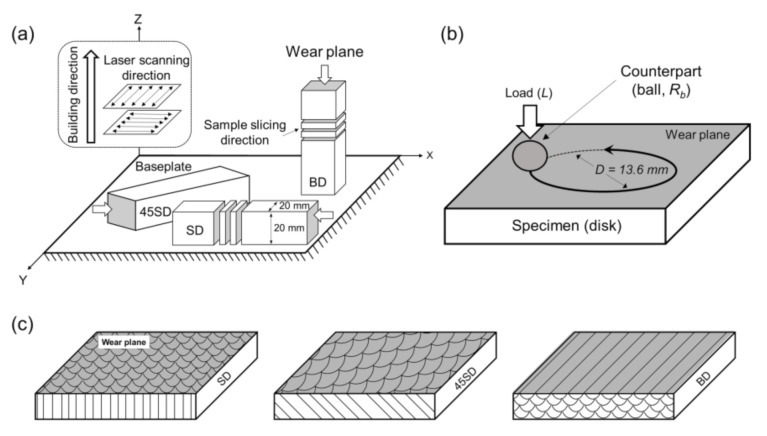
(**a**) Schematic of sample geometries, LPBF scanning strategy, building direction, and wear test directions, (**b**) schematic of the ball-on-disk wear test system, and (**c**) schematic of each sample and wear plane.

To examine the anisotropy of the wear behavior of the samples, three types of samples were built along with three directions with the LPBF building axis. The samples were then sliced in 20 × 20 × 3 mm^3^ blocks for the wear tests, as shown in Figure 1a. The wear tests were performed in the vertical and parallel directions to the baseplate (Figure 1a). The samples with wear planes at 0° and 45° to the laser-scanning direction were designated as SD and 45SD, respectively, whereas the samples with a wear plane vertical to the building direction were designated as BD. The heat-treated SD, 45SD, and BD samples were marked as SD-HT, 45SD-HT, and BD-HT, respectively.

The density was measured as the average of 5 test results of each sample using the Archimedes method. The Vickers microhardness was measured as the average of ten tests on the surface of each specimen under a load of 3 N (HM-220A, Mitytoyo Corp., Kawasaki, Japan). The microstructures were investigated using optical microscopy (OM, Eclipse E200, Nikon, Tokyo, Japan) and field-emission scanning electron microscopy (FE-SEM, JSM-7200F, Jeol Inc., Tokyo, Japan). The wear resistance of the specimens was evaluated using a ball-on-disk testing method machine (Tribometer, J&L Tech, Ansan, Korea), as shown in Figure 1b. All samples were mechanically ground and polished down to 1 μm before the tests, using a diamond suspension. The wear tests were conducted at 25 °C under three normal loads of 5, 20, and 50 N and a constant linear sliding speed of 50 mm/s for a constant sliding distance of 90 m with an AISI 52100 high-carbon-steel ball and ZrO_2_ ball as the counterpart material. The radius of the counter material ball, *R_b_*, was 2.78 mm. The diameter of the wear track, D, was 13.6 mm. Table 3 lists the physical properties of the counterpart materials used in this study. For the given loading conditions of 5, 20, and 50 N, the maximum Hertzian contact stresses for the AISI 52100 steel ball were 627, 994, and 1350 MPa, respectively. The maximum Hertzian contact stresses for the ZrO_2_ ball were 684, 1085, and 1472 MPa, respectively, and wear losses of the specimens and the counterpart balls were measured by weighing them before and after each wear test. The wear rate, λ, was calculated from the volume loss using the following formula:

(1) $$\lambda =\frac{\Delta V}{L}=\frac{\Delta m}{\rho \cdot L}$$

where ∆*V* is the weight volume loss, ∆*m* is the weight loss, *ρ* is the true density of the sample, and *L* is the sliding distance. The weight loss and density were used to calculate the volume loss.

The worn surfaces and the morphology of the wear debris were investigated by FE-SEM. The wear width and depth were measured using a 3D profiler system (3D optical profiler, Contour GT-X, Bruker ASX Pte Ltd., Billerica, MA, USA). The coefficient of friction (COF) versus time was recorded during each wear test.

## 3. Results

### 3.1. Density, Hardness and Microstructure

The true density of the sample was measured to be 7.93 g/cm^3^. The relative density accordingly is approximately 99.1%. No difference in true density was observed for the specimens with different building directions.

Table 4 lists the averaged Vickers microhardness values of the samples used in the wear experiments. The maraging steel produced by the LPBF presented a hardness of ~340–350 Hv in as-built state and 570–590 Hv (i.e., ~1.7 times higher than in the as-built) after HT850 heat treatment. These results can be easily explained by the precipitation strengthening mechanism, i.e., a uniform distribution of fine Ni-rich intermetallic precipitates during the aging of a ductile, low-carbon martensite structure [12,17,22,23,24]. The hardness variations among the three directions are small and within statistical deviations.

**Figure 2:**
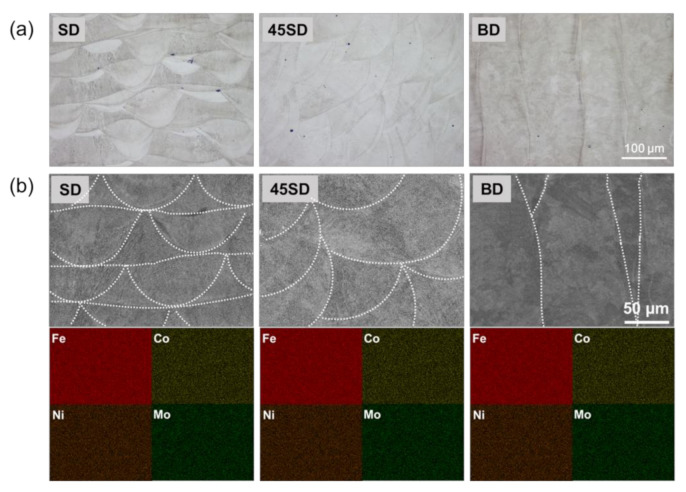
(**a**) OM images (×200) and (**b**) SEM images (×500) and EDS mappings of the wear plane of each as-built sample.

The microstructures of the three different wear planes of the as-built sample were investigated and the results are shown in Figure 2. Figure 2a displays the microstructure observed with the OM. The melt pool boundaries can be clearly observed. The width and height of the bead were approximately 150 and 40 μm, respectively, which were equal to the lamination thickness used during the LPBF process. The EDS analysis was performed for the samples manufactured by the LPBF process to examine the chemical homogeneity. The results are shown in Figure 2b along with corresponding SEM images. The chemical homogeneity was clearly observed in all samples and no differences were observed depending on the building direction. In addition, the beads generated during the LPBF process are observed in the wear plane of each specimen (white line). Further details regarding the microstructure, and tensile behavior of the LPBF-processed maraging steels used in this study can be also found in [22].

### 3.2. Building Direction Dependency of Wear Rates

**Figure 3:**
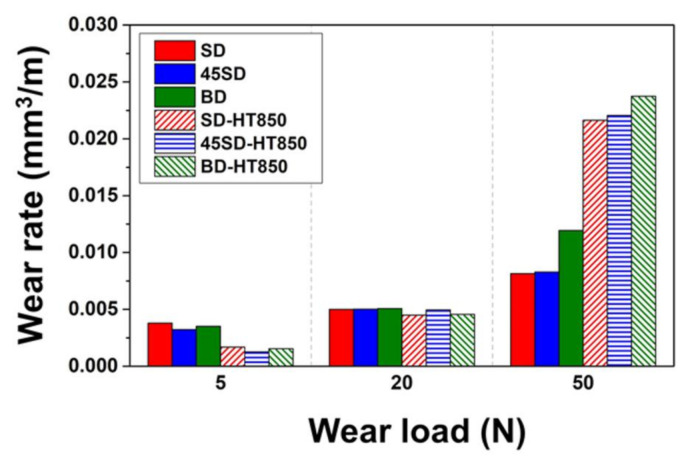
Comparison of wear rates after the wear tests with a total wear distance of 90 m under various wear loads using the AISI 52100 steel for the counterpart material.

Figure 3 shows the differences in the wear rates between the as-built and HT850 samples under the various wear loads. Both the as-built and HT850 samples do not show a pronounced building direction dependency under the wear load of 5 and 20 N. When the wear load was 50 N, both the as-built and the HT850 samples showed strongly anisotropic wear behavior, i.e., wear rates that strongly depend on the wear loading direction with the LPBF building direction. For as-built specimens, more severe wear occurred in the BD specimen than in the SD and 45SD specimens. As shown in the previous study [22], the yield strength of the maraging steel is the weakest in the BD direction among the three directions tested in both as-built and heat-treated states. Thus, the slightly higher wear rates in the BD specimens can be explained by the more severe plastic deformation by wear loading in this direction than in the other two directions tested. This assumption is supported by the fact that pronounced building direction dependency is observed only when the applied wear load is the highest.

At the wear load of 5 N, the HT850 samples exhibited ~50% of the wear rate of the as-built samples in all directions because of increased hardness. When the wear load increased to 20 N, the difference in the wear rate between the as-built and HT850 samples drastically decreased. For the wear load of 50 N, despite the higher hardness, the HT850 samples showed more severe wear than the as-built samples. This seemingly counter-intuitive wear behavior at the high wear load is considered to be from the third body wear by the particles formed from the wear of the counterpart ball. As will be shown in the following sections, the wear of the counterpart AISI 52100 bearing steel ball occurs much more severely for the HT850 samples than the as-built samples.

### 3.3. Wear Mechanism

**Figure 4:**
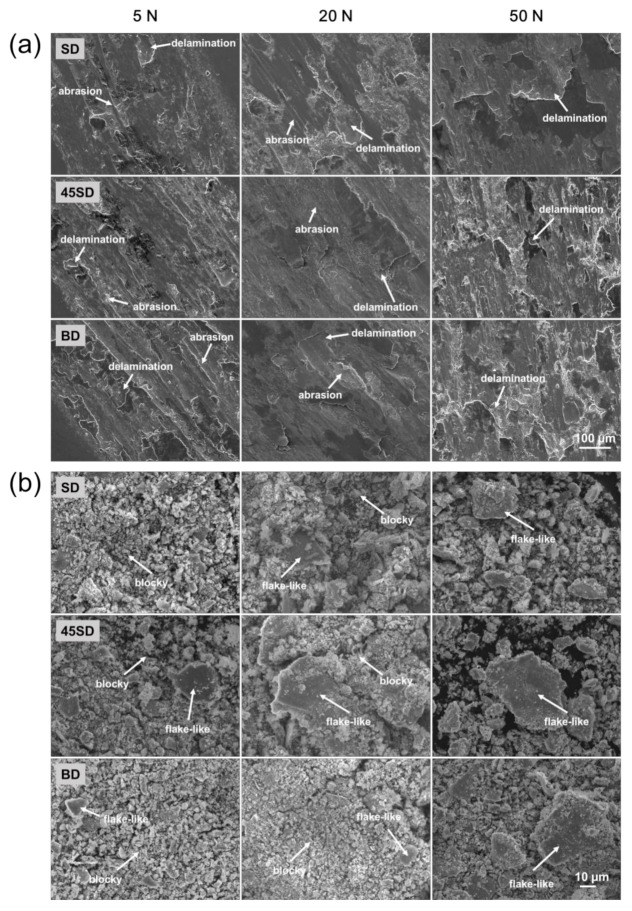
SEM images of (**a**) worn surface and (**b**) wear debris of as-built samples worn against AISI 52100 steel ball with different wear loads.

**Figure 5:**
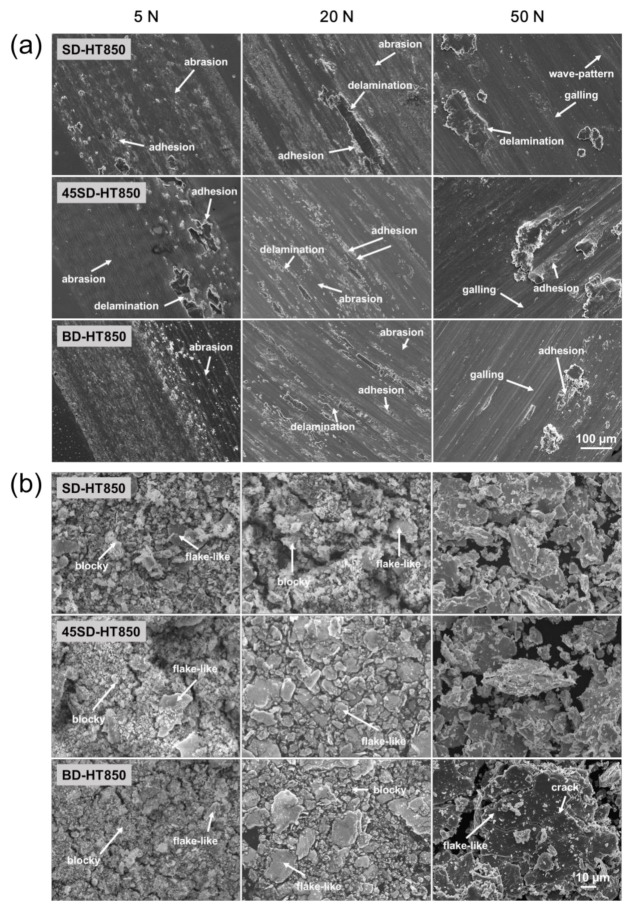
SEM images of (**a**) worn surface and (**b**) wear debris of HT-850 samples worn against AISI 52100 steel ball with different wear loads.

To accurately analyze the wear mechanisms as per the wear conditions, Figure 4 and Figure 5 show the SEM images of worn surface and debris, respectively. Figure 4 shows worn surfaces and debris as per the various wear loads of the as-built sample. At the wear load of 5 N, worn surface morphologies indicated mixed abrasive and adhesive wear mechanisms are observed, as shown in Figure 4a. As shown in Figure 4b, under this wear loading condition, certain flake-like debris along with the blocky debris were reported. The fine blocky debris is believed to be formed by the abrasive wear of the maraging sample, whereas the flake-like debris originates possibly from the delamination of the locally strain-hardened thin film. These results are observed equally in every three-building direction.

With increase in wear load, the delamination marks became larger and the size of the flake-like debris gradually increased, as shown in Figure 4. This behavior can be understood by increasing the depth of the locally strain-hardened film by increasing the wear load. The thicker the hardened film, the easier the delamination damage occurs; consequently, the wear rate will be increased.

In the worn surfaces and wear debris morphologies, no clear difference in wear mechanism as per the building direction is observed. Thus, it can be that the same wear mechanism is active for the three directions tested.

Figure 5 shows the worn surfaces and debris of HT850 samples. At the wear load of 5 N, abrasive and adhesive worn surfaces are observed together in all specimens, as shown in Figure 5a. The higher hardness of the HT850 sample than the as-built sample promotes adhesive wear of the counterpart-bearing steel balls. The worn surfaces showed marks as per the delamination fractures. The wear scars from the delamination fractures in the HT850 samples were smaller than those of the as-built samples, indicating that the delamination followed by the formation of a locally strain-hardened thin film is less pronounced for the heat-treated samples due to their high hardness. As shown in Figure 5b, the wear debris primarily comprise fine and blocky wear debris, which is produced by abrasive wear. Moreover, there is a small amount of flake-type wear debris because of the delamination fractures. For the wear load of 5 N, the morphology of the wear debris of all samples is similar, indicating that the same wear mechanism was active regardless of the sample building direction and the wear load.

Figure 5a shows the worn surfaces reveal the increasing adhesive wear action with increase in wear load. At a 50 N wear load, the worn surface indicates that the wear is dominated by adhesive and delamination fracture without significant abrasive wear damage. Furthermore, marks from galling damage are observed in this condition.

Galling is a severe form of adhesive wear that can occur under high contact pressure [25,26]. The galling wear is accompanied together with severe plastic deformation near the worn surface. Therefore, a relatively large flake-like debris is formed, as can be seen in Figure 5b. Galling wear is observed more clearly on the worn surface of the BD-HT850 sample than can be observed on that of the SD-HT850 and the 45SD-HT850 samples. Figure 4b shows that the size of the flake-like debris is much larger in the case of BD-HT850 than in the other two directions. These results can be understood because the BD-HT850 sample has the lowest yield and tensile strength among the three types of HT850 samples [22]. Therefore, the local plastic deformation of the BD-HT850 sample would be more severe than the other two samples, resulting in a higher wear rate and larger flake-like debris because of the easier delamination fracture.

**Figure 6:**
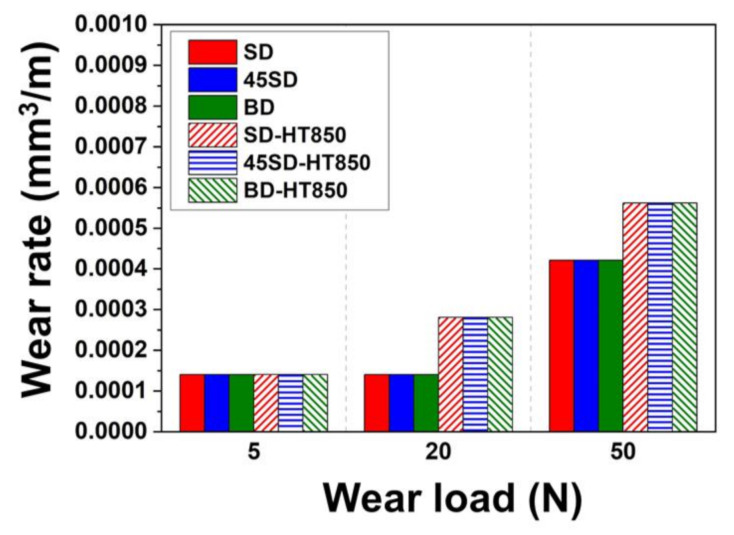
Comparison of the wear rate of AISI 52100 steel counterpart balls.

Figure 6 shows the comparison results of the wear rates of the AISI 52100 bearing steel counter balls for as-built and HT850 samples. There was no dependence on the building direction of the wear rate of the bearing steel counterpart balls. The wear rate of the ball increased with an increased wear load. The wear of the ball was more severe when the maraging sample was heat-treated, which supports the assumption that the third body wear by the particles formed from the wear of the counterpart ball is the primary reason for the faster wear rate in the heat-treated maraging steel compared to that of the as-built state, when the wear load was 50 N.

**Figure 7:**
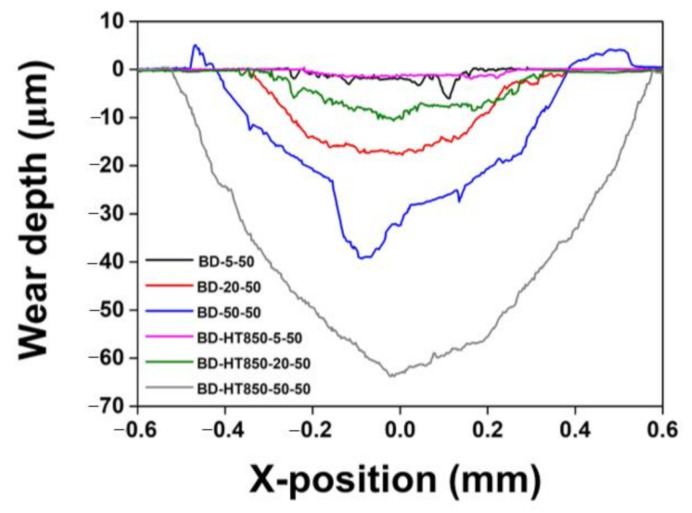
Comparisons of wear track profiles between as-built and HT850 samples worn under various wear loads.

Figure 7 shows the comparison results of the wear depth and width of BD samples for the as-built and HT850 samples. Overall, the worn surface profile results show the same tendency as the wear rates shown in Figure 2. At wear loads of 5 N, the wear depth of the BD sample is deeper than that of the BD-HT850 sample. For the wear loads of 5 and 20 N, there was no significant difference in the width of the wear tracks between the as-built and HT850 samples. For the wear load of 50 N, however, the wear track width of the HT850 sample was pronouncedly deeper than that of the as-built sample, which can be associated with the severe wear of the steel ball for this wear loading condition when the maraging steel sample was heat treated. Owing to the progression of wear on the steel ball, the contact area of the steel ball became wider; eventually, the area that touched the maraging steel sample increased, resulting in a wider wear area of the sample [27].

### 3.4. Evolution of COF for the Bearing Steel Counterpart

**Figure 8:**
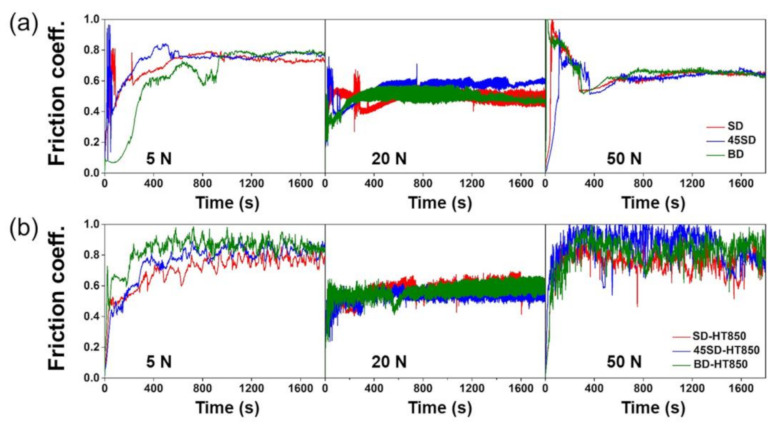
Evaluation of COF with time during wear tests against AISI 52100 bearing steel ball of samples in (**a**) as-built and (**b**) HT850 heat treatment.

Figure 8 shows the evolution of the COFs with time during the wear tests against AISI 52100 bearing steel balls. After the initial period where the sample and ball were adapted to each other, the as-built samples exhibited stable COF evolutions until the test was completed in all wear load conditions, as shown in Figure 8a. There was no clear difference in the stable COF values between the samples SD, 45SD, and BD, indicating that the COF does not depend on the LPBF building direction. At the wear load of 5 N, the COF soared at the beginning of the wear and then stabilized at a value of ~0.7–0.8. The stable COF values for the wear loads of 20 and 50 N were ~0.4–0.6.

For HT850 samples, the COFs evolved similarly to those of the as-built samples for the wear loads of 5 and 20 N. However, for the wear load of 50 N, evolutions of the COFs were slightly different from the as-built samples, as shown in Figure 8b. The COFs evolved relatively unstable and had an amplitude that was greater than the as-built sample. This is probably attributed to the progressive wear of the bearing steel ball during the wear tests.

### 3.5. Effect of Counterpart Materials and Heat Treatment Condition

**Figure 9:**
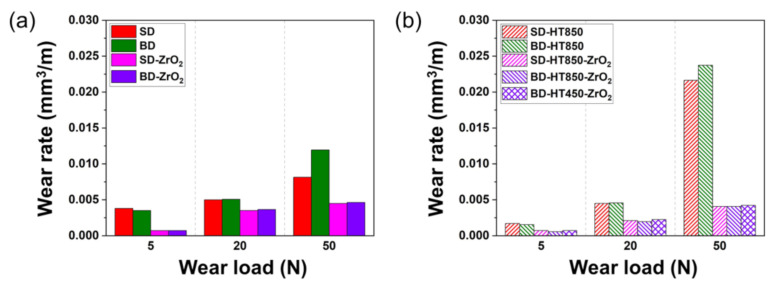
Comparison of the wear rates after the wear tests using AISI 52100 steel and ZrO_2_ balls as counterpart materials. (**a**) As-built and (**b**) heat-treated samples.

Figure 9 shows the wear rates of the maraging steel using the bearing steel and ZrO_2_ balls as the counterpart materials. Because there was almost no difference in the wear rates between the SD and 45SD samples when they wore against the bearing steel, the wear tests against the ZrO_2_ balls were conducted by the SD and BD samples. The influence of direct aging on wear resistance was investigated by comparing the wear rates of the BD-HT850 and BD-HT450 samples worn against the ZrO_2_ balls.

Figure 9 shows the comparison of the wear rates between two counterpart balls show that the wear rates of the maraging steel were much lower when the ZrO_2_ ball was used as the counterpart material rather than the bearing steel ball. This behavior was observed for the maraging steel samples in both the as-built and heat-treated states. Moreover, the wear rates of the heat-treated samples were slower than the as-built samples in every condition tested when the ZrO_2_ ball was used as the counterpart material.

When comparing the wear rates of the BD-HT850 and BD-HT450 samples, the wear rate of the BD-HT450 was slightly faster than the BD-HT850. However, the difference was small, i.e., the maximum difference was <0.00365 mm^3^/m. This indicates that direct aging rather than standard aging treatment followed by the solution can be used as an alternative post-heat treatment conditions for tribological applications of the maraging steels produced by the LPBF.

When the bearing steel ball was used as the counterpart material, anisotropic wear resistance as per the building direction was shown for the wear load of 50 N, for both the as-built and heat-treated states. However, when the ZrO_2_ ball was used as the counterpart material, no clear building direction dependence on the wear rate was reported for every condition tested.

**Figure 10:**
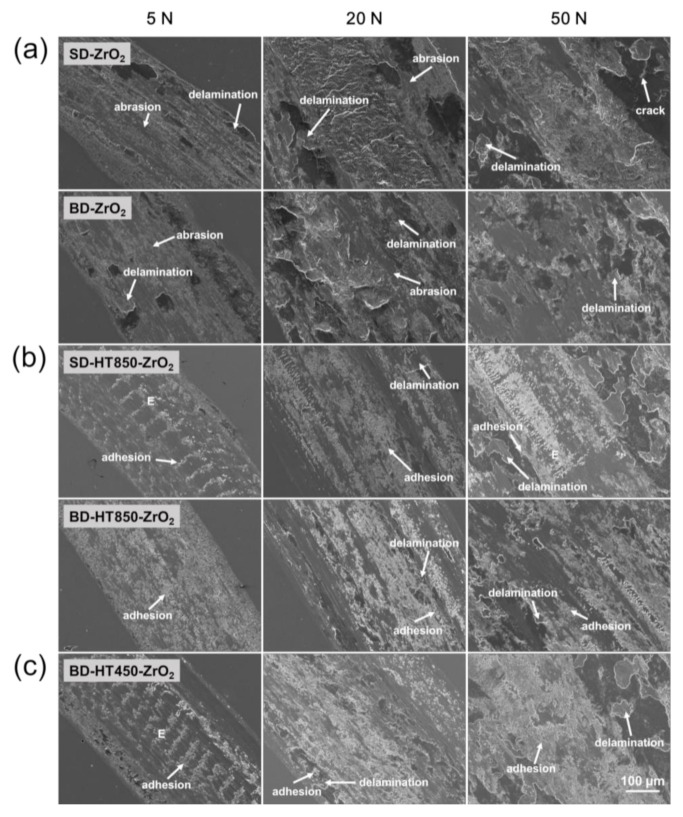
SEM images of the worn surface of maraging steel worn against ZrO_2_ balls: (**a**) as-built, (**b**) HT850 and (**c**) HT450 heat-treated samples.

**Figure 11:**
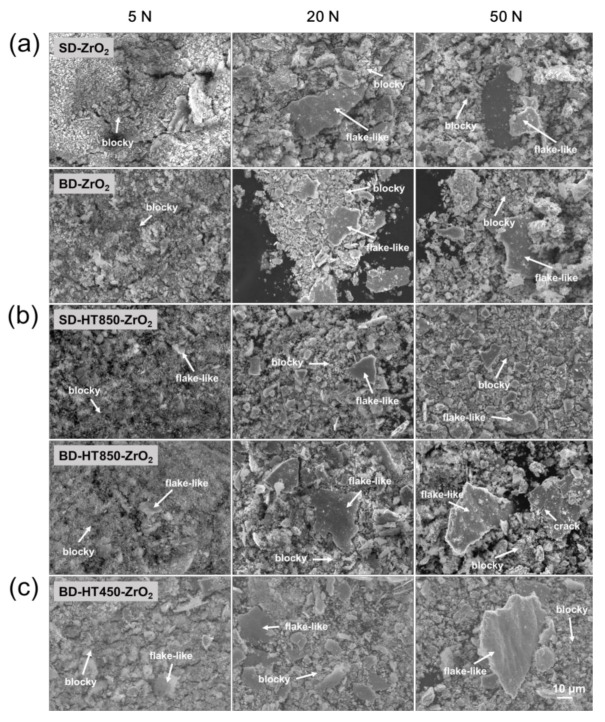
SEM images of the debris of maraging steel worn against ZrO_2_ balls: (**a**) as-built, (**b**) HT850 and (**c**) HT450 heat-treated samples.

Figure 10 and Figure 11 show the worn surfaces and debris of the maraging steel samples produced by the LPBF after the wear tests against ZrO_2_ balls. In the as-built state, the worn surface morphologies of the maraging steel samples were similar to those worn against the bearing steel balls, as shown in Figure 10a. The wear debris morphologies in these cases were nearly the same as the cases against the bearing steel balls, i.e., fine blocky debris with flake-like delaminated particles, as shown in Figure 11a. Hence, the primary wear mechanisms in these cases are considered to be the same as the cases when the bearing steel ball was used as the counterpart material, i.e., severe abrasive wear with delamination fracture of a work-hardened thin layer. For heat-treated samples, the worn surfaces reveal that the dominant mechanism is severe adhesive wear. Furthermore, patterns indicative of fine particles embedded in the worn surface are observed (marked as “E” in Figure 10b,c). The formation of such a pattern is evidence of hard ceramic debris in the worn surface during the wear test and adhesive wear occurs in these areas [28,29]. Therefore, these patterns probably are from a fine and hard ZrO_2_ debris formation during the wear tests. Delamination fractures are observed on the worn surfaces of the heat-treated samples, revealing the local plastic deformation and delamination fracture, followed by work-hardening occur in these cases.

There was no distinguishable difference in the morphology between the worn surfaces of the HT850 and HT450 samples. Therefore, the dominant wear mechanism of the heat-treated maraging steel produced by the LPBF seems not to be influenced by the solution treatment.

**Figure 12:**
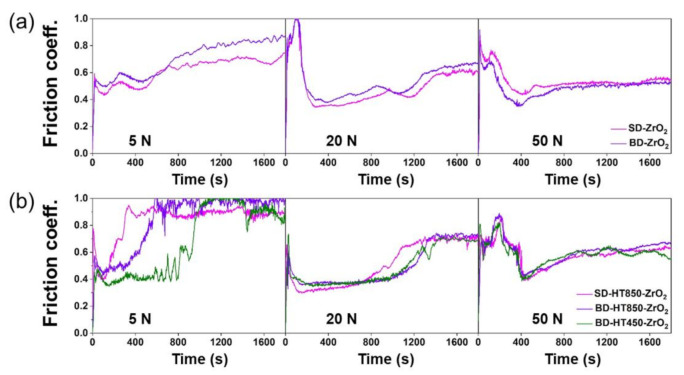
Evolutions of COF with time during the wear test using ZrO_2_ ball as counterpart material: (**a**) as-built and (**b**) heat-treated states.

Figure 12 shows the evolutions of the COFs during the wear tests of the as-built and post heat-treated maraging steel samples produced by the LPBF when the ZrO_2_ balls were used as the counterpart material. The graphs indicate much more stable evolutions of the COFs with far less amplitude compared to the COF evolution when the bearing steel balls were used as the counterpart material. One possible reason for these stable COF evolutions is the ZrO_2_ ball being less worn out during the wear tests. At the wear load of 5 N, the COFs tend to increase gradually until the end of the wear test. This phenomenon is considered because of the slow and progressive adhesive wear action of the ZrO_2_ as the wear sliding slowly occurs. For wear loads of 20 and 50 N, the COFs rise rapidly at the early stages, stabilized, and then gradually increased again during the wear tests. The rapid rise of the COF at the beginning of the wear test is considered to be attributed to the abrasive wear action by the narrow contact area between the ball and the flat surface. The second gradual increases in the COFs are possible because of the formation of the adhesive hard ZrO_2_ particles on the worn surface of the maraging steels. The stable COF values were ~0.4–0.5, which were slightly smaller than those observed from the bearing steel balls.

The evolutions of the COFs of the HT850 and HT450 samples were similar in every wear condition tested. The values of the stable COFs were nearly the same. The evolution of the COFs with the observations of the worn surfaces and debris indicates that direct aging without solution treatment will not cause any major problems under these wear conditions.

The wear test results shown in this study demonstrate that the maraging steel produced by the LPBF exhibits excellent wear resistance to the ZrO_2_ ceramic balls compared to that against the bearing steel ball. Although the hardness of the maraging steel is much less than that of the bearing steel and ZrO_2_, i.e., two counterpart materials used in this study, the wear resistance of the maraging steel to harder ZrO_2_ ball was superior to that against the bearing steel ball. The microstructural investigation and the COF evolutions show that the wear of the ZrO_2_ ball occurred by a limited amount through the adhesion, whereas the bearing steel ball exhibited comparably more severe wear because of the abrasive wear action.

## 4. Summary

In this work, the wear behavior of maraging 18Ni-300 steel fabricated by the LPBF was examined. The building direction dependency of the wear resistance, the influences of heat treatment conditions, and counterpart materials were analyzed under various wear loads in the range of 5 to 50 N. The primary conclusions can be summarized as follows:

1. When the bearing steel ball was used as a counterpart material, the wear resistance of the aging heat-treated maraging steel sample showed superior wear resistance than in the as-built state for the low wear load of 5 N. However, the wear rates of the aging heat-treated sample increased more rapidly with an increase in the wear load compared to those in the as-built state. Consequently, the wear rates of the aging heat-treated sample at the wear load of 20 N were comparable to those in the as-built sample. With further increase in the wear load to 50 N, the wear rates of the aging treated sample became much higher than in the as-built state. This behavior is considered to be from the severe third body wear in the aging heat-treated samples under the high wear load because of the wear debris from the counterpart balls.
2. There was a pronounced building direction dependency of the wear resistance when the wear load was 50 N and the bearing steel counterpart ball was used for both the as-built and aged samples. The wear resistance in the wear loading direction parallel to the LPBF building direction was significantly lower than the other two directions tested. This is probably attributed to the relatively low yield strength of the maraging steel produced by the LPBF in this direction.
3. The wear rates of the maraging steel samples were considerably lower when the ZrO_2_ ball was used as the counterpart material than when the bearing steel ball was used, probably because of the absence of third body wear because of the higher hardness of the ZrO_2_ ball than that of the bearing steel.
4. At the wear test using the ZrO_2_ ball as a counterpart material in the as-built state, the morphologies of the worn surface and wear debris were similar to those worn against the bearing steel balls. For heat-treated samples, however, the worn surfaces reveal that the dominant mechanism is severe adhesive wear. A pattern in which fine hard ceramic debris formed from the wear of ZrO_2_ ball during the wear test is embedded in the worn surface was observed. No pronounced building direction dependence was observed in this case.
5. The directly aged sample, i.e., aged without solution heat treatment, showed wear performance almost the same as that of the sample that underwent solution heat treatment and aging. Therefore, direct aging treatment will not cause any major problems under these wear conditions and can be used as an alternative post heat treatment for tribological applications of the maraging steels produced by the LPBF.

## Figures and Tables

**Table 1:** Chemical composition of 18Ni-300 maraging steel powder.

Element	Fe	Ni	Co	Mo	Ti	Mn	Al
wt.%	Bal.	17–19	8.5	4.0	0.7	≤0.1	≤0.1

**Table 2:** Parameters used for the selective laser melting of 18Ni-300 maraging steel.

Parameters	Values
Laser power	420 W
Scanning speed	1000 mm/s
Hatch spacing	0.1 mm
Lamination thickness	0.04 mm

**Table 3:** Physical properties of the counter materials.

Counter Materials	R_b_ (mm)	Hardness (Hv)	Elastic Modulus (GPa)	Density (g/cm^3^)
AISI 52100	2.78	848	150	7.81
ZrO_2_	2.78	1300	205	6.05

**Table 4:** Effect of building direction and heat treatment on the hardness of the maraging 18Ni-300 steel produced by LPBF.

Heat Treatment Condition	As-Built			Heat-Treated					
Sample ID	SD	45SD	BD	SD-HT850	45SD-HT850	BD-HT850	SD-HT450	45SD-HT450	BD-HT450
Microhardness (Hv)	340 ± 10	348 ± 5	340 ± 7	588 ± 16	579 ± 14	589 ± 12	587 ± 12	582 ± 11	586 ± 9

## Data Availability

The data presented in this study are available from the corresponding authors, upon reasonable request.
